# Enhancing brain metastasis prediction in non-small cell lung cancer: a deep learning-based segmentation and CT radiomics-based ensemble learning model

**DOI:** 10.1186/s40644-023-00623-1

**Published:** 2024-01-02

**Authors:** Jing Gong, Ting Wang, Zezhou Wang, Xiao Chu, Tingdan Hu, Menglei Li, Weijun Peng, Feng Feng, Tong Tong, Yajia Gu

**Affiliations:** 1 Department of Radiology, Fudan University Shanghai Cancer Center, 270 Dongan Road, Shanghai, 200032 China; 2 Department of Oncology, Shanghai Medical College, Fudan University, Shanghai, 200032 China; 3 Department of Cancer Prevention, Fudan University Shanghai Cancer Center, Shanghai, 200032 China; 4 Shanghai Municipal Hospital Oncological Specialist Alliance, Shanghai, 200032 China; 5 Department of Radiation Oncology, Fudan University Shanghai Cancer Center, Shanghai, 200032 China; 6 Department of Medical Imaging, Nantong Tumor Hospital, Nantong University, Nantong, 226361 China

**Keywords:** Non-small cell Lung cancer, Brain Metastasis, CT radiomics, Deep learning, Ensemble learning

## Abstract

**Background:**

Brain metastasis (BM) is most common in non-small cell lung cancer (NSCLC) patients. This study aims to enhance BM risk prediction within three years for advanced NSCLC patients by using a deep learning-based segmentation and computed tomography (CT) radiomics-based ensemble learning model.

**Methods:**

This retrospective study included 602 stage IIIA-IVB NSCLC patients, 309 BM patients and 293 non-BM patients, from two centers. Patients were divided into a training cohort (N = 376), an internal validation cohort (N = 161) and an external validation cohort (N = 65). Lung tumors were first segmented by using a three-dimensional (3D) deep residual U-Net network. Then, a total of 1106 radiomics features were computed by using pretreatment lung CT images to decode the imaging phenotypes of primary lung cancer. To reduce the dimensionality of the radiomics features, recursive feature elimination configured with the least absolute shrinkage and selection operator (LASSO) regularization method was applied to select the optimal image features after removing the low-variance features. An ensemble learning algorithm of the extreme gradient boosting (XGBoost) classifier was used to train and build a prediction model by fusing radiomics features and clinical features. Finally, Kaplan‒Meier (KM) survival analysis was used to evaluate the prognostic value of the prediction score generated by the radiomics–clinical model.

**Results:**

The fused model achieved area under the receiver operating characteristic curve values of 0.91 ± 0.01, 0.89 ± 0.02 and 0.85 ± 0.05 on the training and two validation cohorts, respectively. Through KM survival analysis, the risk score generated by our model achieved a significant prognostic value for BM-free survival (BMFS) and overall survival (OS) in the two cohorts (P < 0.05).

**Conclusions:**

Our results demonstrated that (1) the fusion of radiomics and clinical features can improve the prediction performance in predicting BM risk, (2) the radiomics model generates higher performance than the clinical model, and (3) the radiomics-clinical fusion model has prognostic value in predicting the BMFS and OS of NSCLC patients.

## Introduction

Lung cancer is the leading cause of cancer-related deaths worldwide [1]. Non-small cell lung cancer (NSCLC) accounts for approximately 85% of lung cancer cases [2]. It is estimated that 30-54% of NSCLC patients will suffer brain metastases (BM) at some points during the course of their illness [3]. BM can cause a range of neurological symptoms, including headaches, seizures, and changes in mood or behavior, and can significantly impact a patient’s quality of life. The BM status of advanced NSCLC patients may influence the treatment efficacy of chemotherapy, immunotherapy, and radiotherapy [4–7]. Previous studies have proven that prophylactic cranial irradiation (PCI) is an effective way to prevent the morbidity associated with BM in NSCLC patients [8]. Although PCI can reduce the occurrence rate of BM by approximately 50%, it fails to improve overall survival (OS) of NSCLC patients. PCI is an effective strategy for preventing BM in some NSCLC patients, but it is not appropriate for all patients. Thus, it is necessary to develop a predictor or predictive model to predict the BM risk of advanced NSCLC patients to identify potential patients who may benefit from PCI.

To predict the BM risk of NSCLC patients, the value of clinical features in predicting the BM status of NSCLC patients has been investigated in several studies [9]. For example, Zhang F et al. developed a nomogram to predict 3- and 5-year BM rates by using four clinical factors, namely, neuron-specific enolase, histological type, number of metastatic lymph nodes, and tumor grade [10]. The results showed that a clinical factor-based nomogram can be used to predict BM status for NSCLC patients. Although baseline clinical characteristics are associated with BM occurrence in NSCLC patients, the predictive performance is limited.

Meanwhile, numerous studies have investigated computed tomography (CT) image-based radiomics features to predict the occurrence of BM [11]. The radiomics model consists of a few procedures, i.e., tumor segmentation, quantitative imaging feature extraction, feature selection, and classifier training/testing [12–15]. Sun F et al. developed a CT radiomics and clinical integrated nomogram to predict the occurrence of BM for curatively resected locally advanced NSCLC (LA-NSCLC) patients [16]. The results indicated that the prediction performance in terms of predicting BM-free survival in curatively resected LA-NSCLC patients can be improved by integrating CT radiomics and clinical features. However, another study provided an in-depth comparative analysis of CT-based radiomics and clinical factors and showed that CT-based radiomics features of primary NSCLC cannot improve the predictive efficiency of a clinical risk factor (age and adenocarcinoma histology)-based model for BM development in radically treated stage III NSCLC patients [8]. The contradictory results yielded by these studies may be caused by the different populations of enrolled patients. Despite the different efficiencies of the radiomics-clinical integrated model, CT-based radiomics features can be applied to predict the BM status of NSCLC patients.

Although CT-based radiomics has been validated in predicting BM status for locally advanced or radically treated stage III NSCLC patients, whether it can be used in predicting advanced NSCLC patients who may develop BM and thus benefit from PCI has not yet been investigated [17]. In addition, how to integrate CT-based radiomics features and clinical features to improve the performance of the model also needs to be explored [18].

**Fig. 1:**
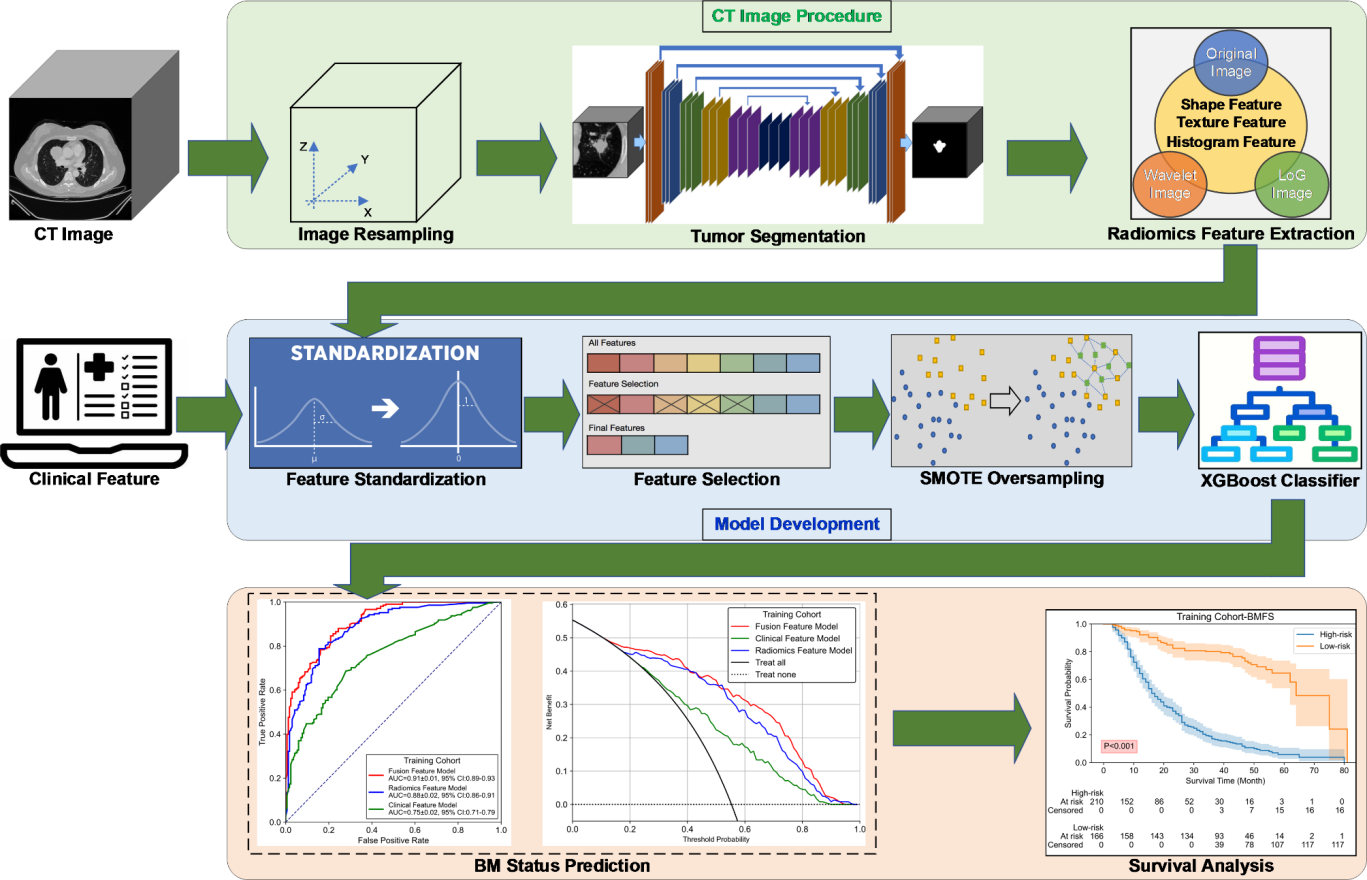
Workflow of the proposed BM prediction model

To address these challenges, we propose a novel approach for predicting BM in NSCLC patients using CT radiomics-based ensemble learning. Figure 1 illustrates the workflow of the proposed BM prediction model. This approach fuses CT-based radiomics and clinical features to train and build extreme gradient boosting (XGBoost)-based ensemble models to improve the prediction accuracy and reduce the overfitting risk. Overall, our study highlights the potential of ensemble learning and radiomics-based approaches for improving the accuracy of predicting BM status in advanced NSCLC patients. This approach could have significant implications for the early detection and treatment of this challenging complication.

## Materials and methods

### Dataset

We retrospectively collected data from 602 advanced (stage III-IV) NSCLC patients from two centers. Among them, 537 patients were enrolled from Fudan University Shanghai Cancer Center (FUSCC) between April 2015 and May 2019. The other 65 patients were recruited from Nantong Tumor Hospital (NTH) between April 2016 and August 2020. The inclusion criteria were as follows: (1) histopathologically diagnosed with NSCLC; (2) clinically diagnosed with stage III-IV NSCLC based on the 8th edition of the TNM staging system; (3) underwent baseline contrast-enhanced CT within one week before surgery or biopsy; (4) had no evidence of BM before treatment; (5) had no other primary malignant tumor at baseline and during follow-up; and (6) had at least 3 years of subsequent follow-up for BM. The exclusion criteria were as follows: (1) lack of CT scan; (2) poor CT image quality; and (3) lack of clinical information.

The CT scan before treatment and several serum tumor biomarkers (i.e., carcinoembryonic antigen (CEA), cytokeratin 19 fragment (CYFRA21-1), neuron-specific enolase (NSE), and alpha-fetoprotein (AFP)) were collected for each patient. All CT scans were acquired by using a multislice CT scanner (manufacturers: Siemens, Philips, Toshiba or United Imaging Healthcare) with a tube voltage of 120 kVp and 100–300 mA. The pixel spacing of each CT image ranged from 0.62 to 0.98 mm. The slice thickness of each CT scan was in the range of [1 mm, 5 mm]. Each CT slice was reconstructed with an image matrix of 512×512 pixels. The CT images were retrieved from the picture archiving and communication system in digital imaging and communications in medicine (DICOM) format. 70% of the FUSCC patients (376 patients) were randomly selected to develop a training cohort to train the prediction model. The remaining 30% of the FUSCC patients (161 patients) were selected as validation cohort 1 to validate the proposed model. The Nantong Tumor Hospital patients were used as an independent external validation dataset to develop validation cohort 2.

The study was approved by the Ethics Committees of Fudan University Shanghai Cancer Center and Nantong Tumor Hospital, and informed consent was waived because of the retrospective nature of the study.

### Deep residual U-Net based tumor segmentation

We first proposed a deep residual U-Net network to accurately segment lung tumors in CT images. As the resolution of CT images in our dataset was nonuniform, a cubic B-spline interpolation algorithm was implemented to resample all CT images with a new spacing of [1 mm, 1 mm, 1 mm]. The intensity of the CT images was clipped into a range of [-1200, 400] and then transformed to [0, 1]. Each tumor was delineated on CT images by two junior radiologists (T.H. and M.L.) in a slice-by-slice fashion. ITK-Snap software (version 3.8.0, http://www.itksnap.org) was applied to delineate the boundaries of each lung tumor. For cases with multiple lesions, the primary tumor was located by reviewing the histopathological reports in the hospital’s electronic medical record system. Finally, the volume of interest (VOI) of each primary lesion was reconfirmed by a senior radiologist (Y.G.). To evaluate the consistency of VOIs delineated by two junior radiologists, twenty cases were randomly selected to test the segmentation consistency. After testing the VOIs determined by the two junior radiologists, the Dice coefficient was 0.82 ± 0.06, and the Jaccard similarity coefficient was 0.71 ± 0.11. The mask of the delineated VOI was employed as the ground truth to train and build a deep residual U-Net network. To reduce the computational cost, we cropped each tumor into a cubic patch of 96 mm×96 mm×96 mm by referring to the tumor location delineated by the radiologist.

**Fig. 2:**
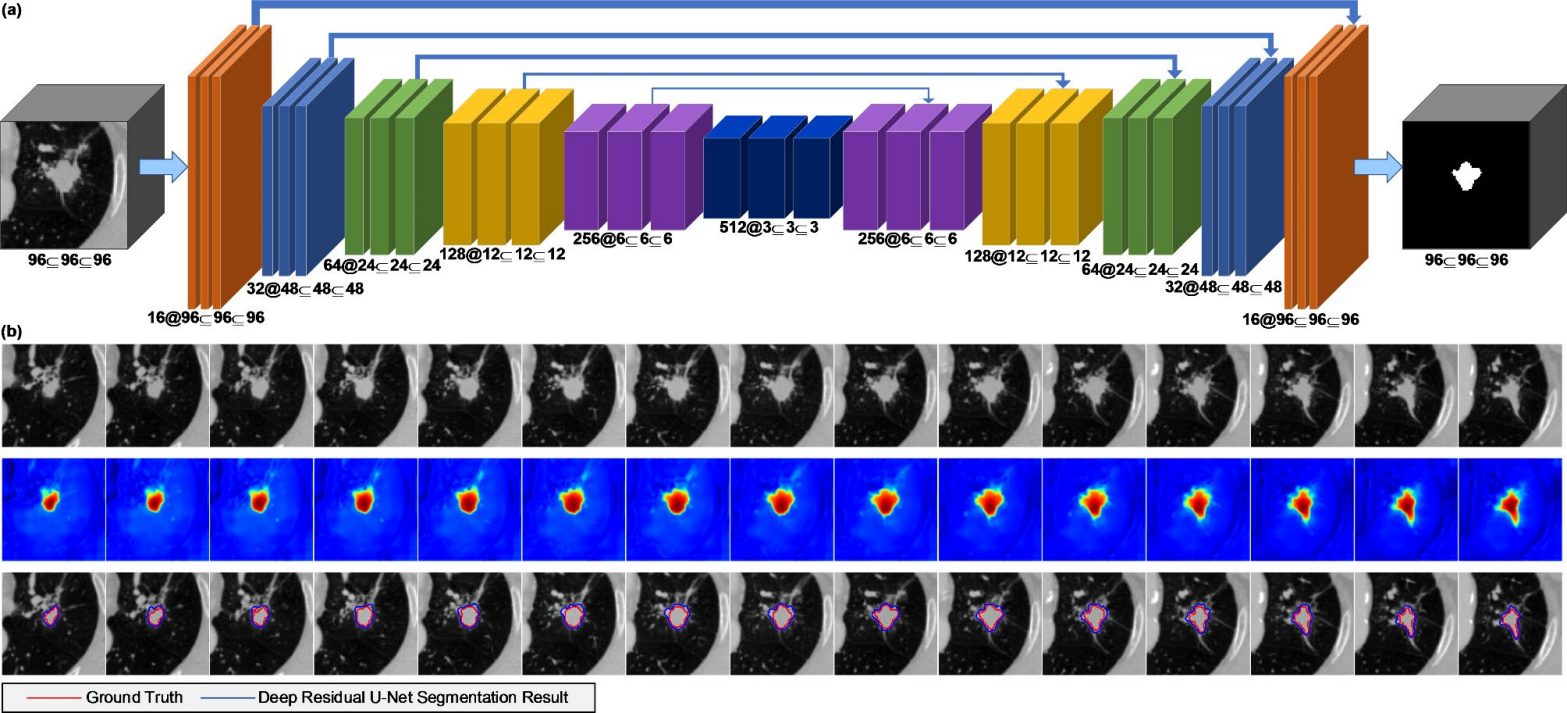
The architecture and segmentation result of the deep residual U-Net network. (**a**) The architecture of the proposed deep residual U-Net network; (**b**) the output heatmap and segmentation result generated by the U-Net model. The images from top to bottom depict the original tumor images, output probability heatmaps of the deep U-Net model, and segmentation results of the proposed model and the ground truth

To segment the lung tumors in the CT images, we employed a 3D residual network as the backbone to construct an “encoder–decoder” architecture U-Net model [19]. The “encoder” path has five submodules, each of which consists of two convolutional layers followed by a rectified linear unit (ReLU). After each submodule, a convolutional layer configured with a kernel size of 1×1×1 and a stride of 2 was used to downsample the image. The “decoder” path also contains five submodules. The resolution was increased successively by upsampling. Finally, the prediction probability is output for each pixel. The network also uses a skip connection to connect the upsampling result to the output of the submodule with the same resolution in the encoder as the input of the next submodule in the decoder path. For the 3D residual U-Net, an input patch size of 96×96×96 and a batch size of 64 are configured to train the model. Figure 2 (a) shows the architecture of the deep residual U-Net-based lung tumor segmentation model.

To train the U-Net model, we applied a series of data augmentation techniques to increase the training data size. These techniques include 90-degree increment rotations, intensity shifts with randomly selected offset, and random flipping [20]. To improve the generalizability of the model, data augmentation was performed on the fly during the training process. The Dice loss was applied to evaluate and optimize the deep residual U-Net model. The formula function of the Dice loss is as follows:

$$DL=1 - 2\frac{{\sum\nolimits_{i}^{N} {{p_i}{g_i}} }}{{\sum\nolimits_{i}^{N} {p_{i}^{2}} +\sum\nolimits_{i}^{N} {g_{i}^{2}} }}$$

where the sum is calculated over N voxels of the predicted binary segmentation volume ${p_i} \in P$ and the ground truth binary volume ${g_i} \in G$. To train the U-Net model, we used adaptive moment estimation (Adam) optimization with a default learning rate of 1 × 10^− 4^ and weight decay of 1 × 10^− 4^.

### Radiomics feature extraction and selection

A total of 1106 radiomics features were computed to decode the imaging phenotypes of each primary lung tumor. The initial feature pool consisted of three types of imaging features: original features, Laplacian of Gaussian (LoG) features, and wavelet features. The LoG and wavelet features were computed based on the transformed image using LoG and wavelet image filters, respectively. The LoG filter was configured with δ values of 1.0, 2.0, and 3.0. Additionally, a 3D wavelet filter was employed to decompose the original image into eight subbands. Each type of image feature included shape features, histogram-based first-order features, and texture features. The open-source Python package PyRadiomics (https://github.com/AIM-Harvard/pyradiomics) was used to extract the radiomics features [21].

To standardize the radiomics features, a zero-mean normalization technique was employed to process each feature type, thereby removing the mean and scaling the features to unit variance. The radiomics features were initially selected by removing features with low variance, and a threshold of 1.0 was set for the feature selector. To further reduce the dimensionality of the radiomics features, a recursive feature elimination (RFE) method was employed and configured with least absolute shrinkage and selection operator (LASSO) regularization [22]. This method was used to select the optimal image features for model development. The feature selector was applied to the training dataset and subsequently transformed into the validation datasets.

### Ensemble learning-based model development

To address the issue of imbalanced datasets and avoid bias in the model development, the synthetic minority oversampling technique (SMOTE) method was employed to resample the minority category [23]. Specifically, the SMOTE was only applied to the training dataset. An ensemble learning algorithm, the XGBoost classifier, was then utilized to train and develop the classification model for predicting the BM status of advanced NSCLC patients. This classifier operates on the principles of gradient boosting, combining the predictions of multiple decision trees to make the final prediction. The XGBoost algorithm employes a sequence of decision trees, with each subsequent tree correcting errors made by its predecessor. The trees are iteratively added, and the algorithm optimizes the weights of each tree to minimize the loss function. The process of adding trees continues until the specified number of trees is reached or until the loss function is no longer improved.

To compare the model predictive performance with different BM status feature types, three distinct classification models were developed, including a CT radiomics feature model, a clinical feature model, and a fused feature model. To mitigate the potential bias caused by the use of varying algorithms, the same feature selection method, sample oversampling technique, and XGBoost classifier were utilized across all three models.

### Statistical analysis

To evaluate the performance of the proposed models, the area under the receiver operating characteristic (ROC) curve (AUC) and the corresponding 95% confidence interval (CI) were calculated. To estimate the 95% CI of the AUC, a bootstrap resampling procedure with 1000 iterations was used. The Delong test was employed to compare the ROC curves of different models. Several quantitative evaluation metrics, including accuracy (ACC), sensitivity (SEN), specificity (SPE), positive predictive value (PPV), negative predictive value (NPV), odds ratio (OR), F1 score, F1_weighted_ score and Matthews correlation coefficient (MCC), were also further computed to assess the model performance. The optimal cutoff threshold of the proposed model was computed by the Youden index. Decision curve analysis (DCA) was used to evaluate and compare the performance of different prediction models in terms of clinical decision-making.

The Kaplan‒Meier (KM) survival analysis method was used to evaluate the prognostic value of the rad-score generated by the fused feature model. In the survival analysis process, the log-rank test was used to compare the groups and determine significant differences between the KM curves. Harrell’s concordance index (C-index) and the hazard ratio (HR) were used to evaluate the value of the rad-score in estimating the BM-free survival (BMFS) and OS. For all results of statistical analysis, P < 0.05 (two-sided tests) was considered significant.

All the model development and statistical analysis processes were implemented in Python (version 3.9, https://www.python.org). Several publicly available python libraries, i.e., PyRadiomics, PyTorch, SimpleITK, scikit-learn, XGBoost, lifelines, SciPy, Matplotlib, NumPy, and Pandas, were applied to develop the classification models.

## Results

### Patient demographics and clinical characteristic

Table 1 summarizes the characteristics of advanced NSCLC patients in the training and validation cohorts. The proportions of BM patients were 55.3% (208/376), 50.9% (82/161) and 29.2% (19/65) in the training cohort, validation cohort 1 and validation cohort 2, respectively. The overall dataset included data from 332 males (55.1%) and 270 females (44.9%). The average age of all patients was 59 (19–83). Among them, 238 (39.5%) patients had a history of smoking, 478 (79.4%) patients had been histopathologically confirmed to have adenocarcinoma, and 92 (15.3%) patients had been confirmed to have squamous cell carcinoma. Four serum tumor biomarkers, CEA, CYFRA21-1, NSE, and AFP, were tested. By testing with a t test, a significant difference in the thickness of the CT image was observed in both the training and validation cohorts.

**Table 1:** Characteristics of advanced NSCLC patients in the training and validation cohorts

Characteristic	Training Cohort		P Value	Validation Cohort1		P Value	Validation Cohort2		P Value
	BM = 208	NonBM = 168		BM = 82	NonBM = 79		BM = 19	NonBM = 46	
Sex			0.41			0.19			0.52
Male	111 (29.52)	96 (25.53)		49 (30.43)	39 (24.22)		12 (18.46)	25 (38.46)	
Female	97 (25.80)	72 (19.15)		33 (20.50)	40 (24.85)		7 (10.77)	21 (32.31)	
Age	57 ± 9.92	58 ± 9.86	0.97	57 ± 9.41	58 ± 11.06	0.61	64.±12.00	63 ± 9.16	0.23
Smoking			0.19			0.36			0.54
Current or Former	79 (21.01)	75 (19.95)		37 (22.98)	30 (18.63)		4 (6.15)	13 (20.00)	
Never	129 (34.31)	93 (24.73)		45 (27.95)	49 (30.44)		15 (23.08)	33 (50.77)	
Pathology			0.5			0.81			0.96
Adenocarcinoma	176 (46.81)	126 (33.51)		63 (39.13)	57 (35.40)		16 (24.62)	40 (61.54)	
Squamous Cell Carcinoma	17 (4.52)	40 (10.64)		9 (5.59)	18 (11.18)		3 (4.62)	5 (7.69)	
Others	15 (3.99)	2 (0.53)		10 (6.21)	4 (2.49)		0 (0.00)	1 (1.54)	
CEA	30.65 ± 83.80	19.15 ± 60.17	0.12	14.32 ± 27.02	10.15 ± 18.41	0.26	53.34 ± 93.58	8.19 ± 14.98	0.003
CYFRA21-1	5.19 ± 8.18	5.98 ± 9.24	0.39	5.09 ± 6.88	4.93 ± 4.00	0.86	6.78 ± 7.15	6.20 ± 15.56	0.84
NSE	11.87 ± 9.03	12.34 ± 5.77	0.56	12.20 ± 8.69	12.03 ± 3.79	0.87	17.60 ± 7.56	13.31 ± 8.94	0.06
AFP	1.58 ± 2.00	1.88 ± 2.43	0.19	1.58 ± 1.60	2.36 ± 4.18	0.13	1.85 ± 1.58	1.54 ± 1.71	0.50
Thickness	1.77 ± 1.46	1.23 ± 0.80	< 0.001	1.36 ± 1.04	1.10 ± 0.47	0.045	1.53 ± 0.94	1.18 ± 0.38	0.04
Spacing	0.75 ± 0.06	0.77 ± 0.06	0.006	0.76 ± 0.06	0.76 ± 0.06	0.57	0.71 ± 0.03	0.69 ± 0.06	0.05

### Tumor segmentation results using deep residual U-Net

When tested on the evaluation cohort, our proposed deep residual U-Net achieved a Dice similarity coefficient of 0.88 ± 0.08, an intersection over union score of 0.79 ± 0.11, a Hausdorff distance of 9.35 ± 6.64, and an average surface distance of 0.85 ± 0.72. Figure 2(b) shows an example of the segmentation results generated by the deep residual U-Net model.

### Optimal radiomics and clinical features selected during model development

**Fig. 3:**
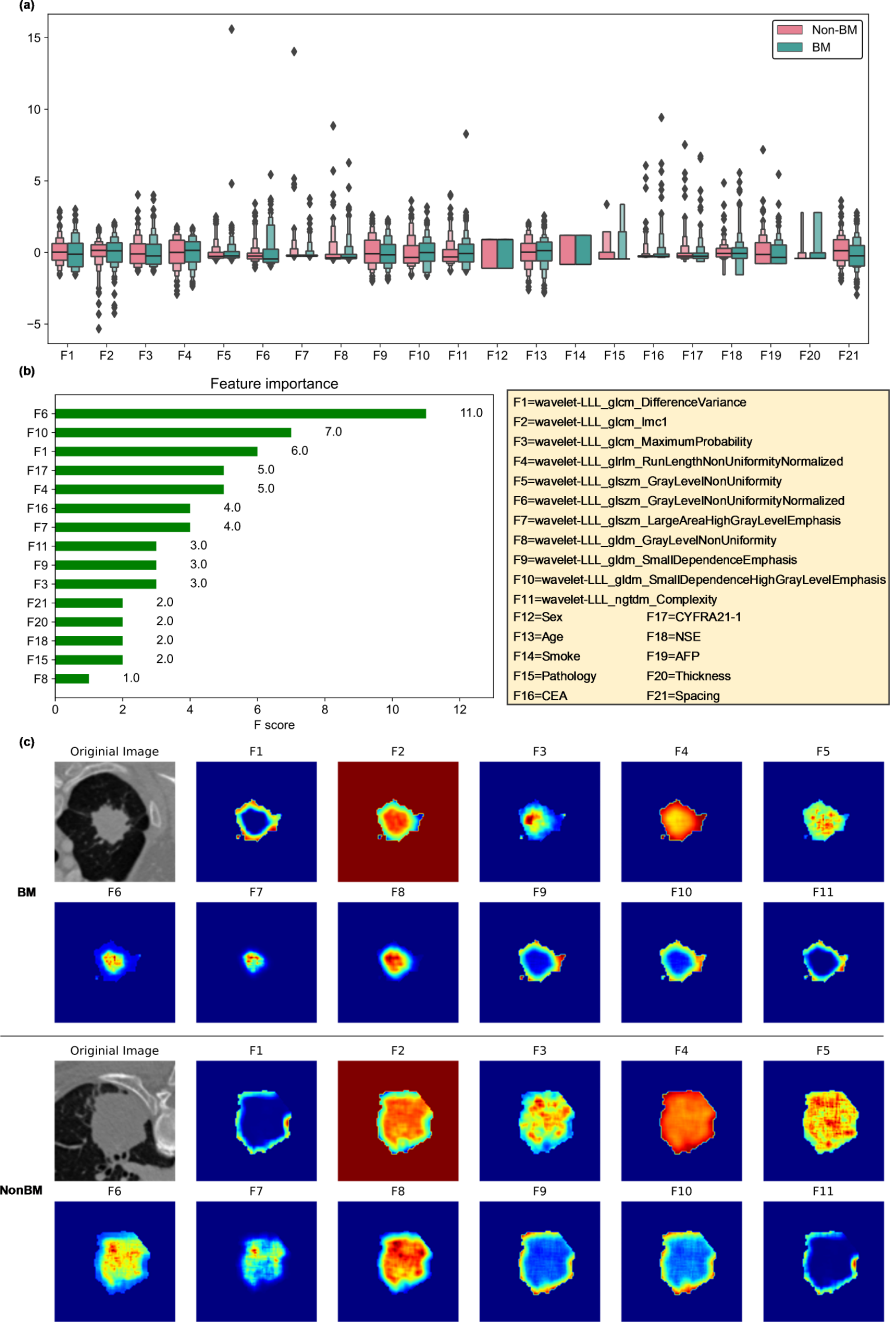
Comparison and visualization of the selected features in the prediction model. (**a**) Boxplots of the selected features; (**b**) feature importance of the XGBoost classifier-based fused feature model; (**c**) examples of the selected radiomics features for BM and non-BM patients by using a voxel-based radiomics feature visualization technique

Figure 3 (a) shows boxplots of the selected features in the fused feature model. A total of 11 wavelet features and 10 clinical features were involved. The 11 wavelet features were texture features, including three gray level cooccurrence matrix features, three gray level dependence matrix texture features, one gray level run length matrix feature, three gray level size zone matrix texture features, and one neighboring gray tone difference matrix texture feature. In the CT radiomics feature model, ten wavelet features involving F2-F11 in Fig. 3(a) were selected. This result showed that wavelet features play a vital role in predicting BM status. Figure 3 (b) lists the feature importance of the XGBoost classifier-based fused feature model. The bar chart shows the relative importance of each selected feature. To further compare and visualize the selected CT radiomics features, Fig. 3 (c) shows a plot containing examples of 11 selected radiomics features for BM and non-BM patients by using a voxel-based radiomics feature visualization technique.

### Comparison of the BM prediction performance

**Fig. 4:**
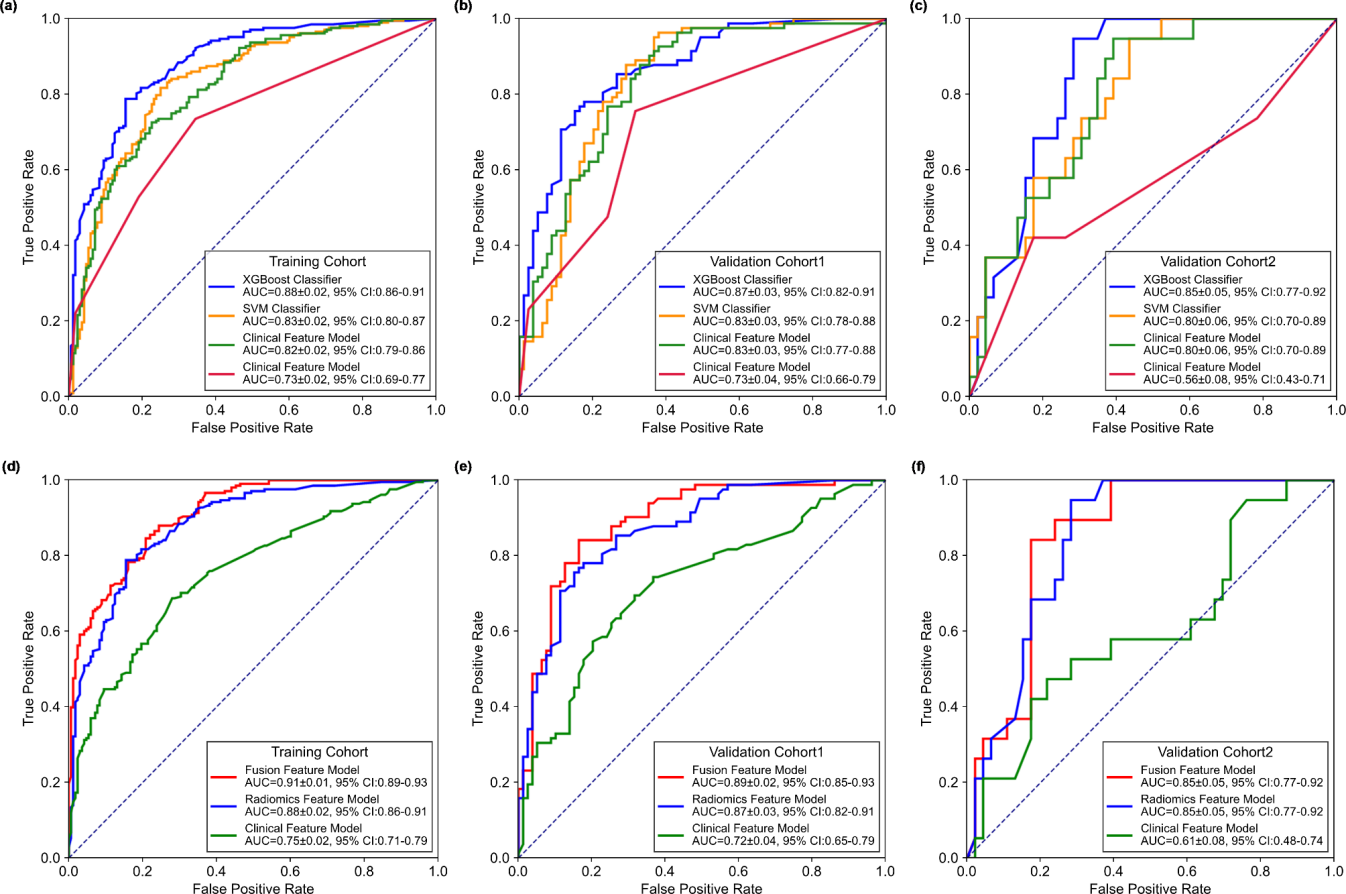
ROC curves of the different models for the training and validation cohorts. (**a**)-(**b**) ROC curves of four different classifiers; (**c**)-(**d**) ROC curves and the corresponding AUC values of the radiomics, clinical and fused feature models, respectively

To compare the performance of different machine learning classifiers, the ROC curves of four classifiers, namely, the XGBoost classifier, support vector machine (SVM) classifier, multilayer perceptron (MLP) classifier, and decision tree classifier, were plotted, as shown in Fig. 4 (a)-(c). Compared with the other three classifiers, the XGBoost classifier achieved the highest AUC values for both the training and validation cohorts (P < 0.05). Table 2 shows a comparison of the ACC, SEN, SEP, PPV, NPV, OR, F1 score, F1_weighted_ score and MCC of the four classifiers for the training and two validation cohorts. The quantitative metrics also indicated the same trend, i.e., the XGBoost classifier achieved the best performance. Thus, the XGBoost classifier was selected to build the prediction model.

**Table 2:** Comparisons of the performance of different classifiers by using radiomics features

Classifier	Dataset	ACC (%)	SEN (%)	SPE (%)	PPV (%)	NPV (%)	OR	F1 Score	F1_Weighted_ Score	MCC
XGBoost	TC	80.59	81.73	79.17	82.93	77.78	17	0.82	0.81	0.61
	VC1	79.5	76.83	82.28	81.82	77.38	15.39	0.79	0.79	0.59
	VC2	76.92	84.21	73.91	57.14	91.89	15.11	0.68	0.78	0.53
SVM	TC	76.60	74.52	79.17	81.58	71.51	11.11	0.78	0.77	0.53
	VC1	77.02	76.83	77.22	77.78	76.25	11.24	0.77	0.77	0.54
	VC2	70.77	68.42	71.74	50.00	84.62	5.50	0.58	0.72	0.37
MLP	TC	74.20	73.08	75.60	78.76	69.40	8.41	0.76	0.74	0.48
	VC1	75.78	76.83	74.68	75.90	75.64	9.78	0.76	0.76	0.52
	VC2	67.69	68.42	67.39	46.43	83.78	4.48	0.55	0.69	0.33
Decision Tree	TC	69.95	73.56	65.48	72.51	66.67	5.28	0.73	0.70	0.39
	VC1	72.05	75.61	68.35	71.26	72.97	6.70	0.73	0.72	0.44
	VC2	64.62	42.11	73.91	40.00	75.56	2.06	0.41	0.65	0.16

TC: Training Cohort; VC1: Validation Cohort1; VC2: Validation Cohort2

**Fig. 5:**
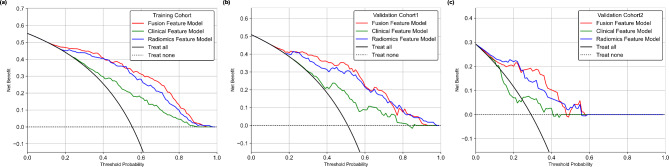
DCA curves of the three models to assess the net benefits

Figure 4 (d)-(f) shows a comparison the ROC curves and the corresponding AUC values of the radiomics feature model, clinical feature model and fused feature model, respectively. Comparing with CT radiomics model, the fusion model yielded AUC values of 0.91 ± 0.01 (95 CI: 0.89–0.93), 0.89 ± 0.02 (95 CI: 0.85–0.93) and 0.85 ± 0.05 (95 CI: 0.77–0.92) for the training cohort (P < 0.05), validation cohort 1 (P < 0.05) and validation cohort 2 (P > 0.05), respectively. The fused feature model achieved significantly higher AUC values than those of the clinical feature model for all cohorts (P < 0.05). Meanwhile, the performance of the CT radiomics feature model was significantly higher than that of the clinical feature model (P < 0.05). Table 3 shows a summary and comparison of the quantitative metrics of the three prediction models. The same trend, i.e., fusing CT radiomics features and clinical features could improve the model performance in term of predicting the BM status of advanced NSCLC patients, was observed. To evaluate the clinical values of the three models, Fig. 5 shows a comparison of the DCA curves of the three models to assess the net benefits. This result indicated that the fused feature model performs better than the radiomics feature model and clinical feature model in terms of clinical usefulness.

**Table 3:** Comparisons of the performance of different prediction models in the training and validation cohorts

Model	Dataset	ACC (%)	SEN (%)	SPE (%)	PPV (%)	NPV (%)	OR	F1 Score	F1_Weighted_ Score	MCC
Radiomics Feature Model	TC	80.59	81.73	79.17	82.93	77.78	17	0.82	0.81	0.61
	VC1	79.5	76.83	82.28	81.82	77.38	15.39	0.79	0.79	0.59
	VC2	76.92	84.21	73.91	57.14	91.89	15.11	0.68	0.78	0.53
Clinical Feature Model	TC	67.02	57.69	78.57	76.92	60	5	0.66	0.67	0.37
	VC1	67.08	52.44	82.28	75.44	62.5	5.12	0.62	0.66	0.36
	VC2	58.46	57.89	58.70	36.67	77.14	1.95	0.45	0.60	0.15
Fusion Feature Model	TC	81.91	84.13	79.17	83.33	80.12	20.15	0.84	0.82	0.63
	VC1	83.23	82.93	83.54	83.95	82.5	24.66	0.83	0.83	0.66
	VC2	80.00	84.21	78.26	61.53	92.31	19.2	0.71	0.81	0.58

TC: Training Cohort; VC1: Validation Cohort1; VC2: Validation Cohort2

### Prognostic evaluation based on the Radiomics–Clinical Fusion Model

**Fig. 6:**
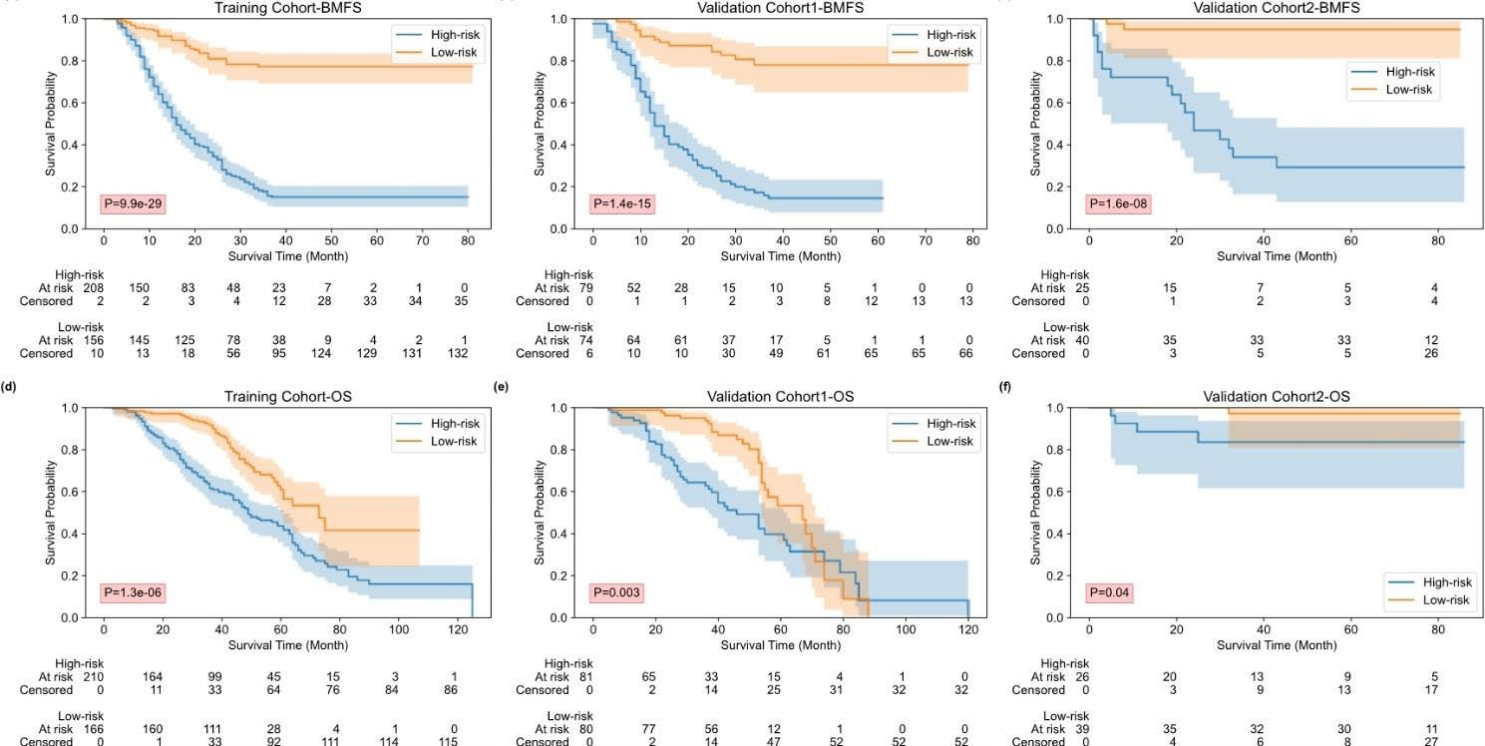
BMFS and OS KM survival curves for the training and validation cohorts in terms of the prediction scores generated by the fused feature model. (**a**)-(**b**) BMFS KM curves for the training and validation cohorts; (**c**)-(**d**) OS KM curves for the training and validation cohorts

Figure 6 shows the BMFS and OS KM survival curves of the training and two validation cohorts for prediction scores generated by the fused feature model. Through the KM survival analysis, the stratification effects of the prediction scores were significant for the training and two validation cohorts in terms of estimating BMFS and OS (all P < 0.05, log-rank test). Table 4 lists the C-index, HR and P value of the fusion model in terms of predicting BMFS and OS for the training and validation cohorts. The signatures constructed based on the binary classification results of the fused feature model had prognostic predictive performance in terms of predicting BMFS and OS for the training cohort (BMFS, HR: 6.40, 95% CI: 4.41–9.31, P < 0.001; OS, HR: 2.22, 95% CI: 1.59–3.08, and P < 0.001), validation cohort 1 (BMFS, HR: 7.60, 95% CI: 4.26–13.59, P < 0.001; OS, HR: 2.00, 95% CI: 1.25–3.21, and P = 0.003) and validation cohort 2 (BMFS, HR: 14.06, 95% CI: 4.07–48.65, P < 0.001; OS, HR: 6.97, 95% CI: 0.78–62.62, and P = 0.04).

**Table 4:** The C-index, HR and P value of the fusion model in terms of predicting BMFS and OS for the training and validation cohorts

	Dataset	C-index	HR (95% CI)	P Value
BMFS	Training Cohort	0.68	6.40 (4.41–9.31)	9.9×10^− 29^
	Validation Cohort1	0.71	7.60 (4.26–13.59)	1.4×10^− 15^
	Validation Cohort2	0.78	14.06 (4.07–48.65)	6.3×10^− 8^
OS	Training Cohort	0.62	2.22 (1.59–3.08)	1.3×10^− 6^
	Validation Cohort1	0.65	2.00 (1.25–3.21)	0.003
	Validation Cohort2	0.73	6.97 (0.78–62.62)	0.04

## Discussion

Accurately predicting the risk of BM is a critical aspect of personalized treatment planning for advanced NSCLC patients to improve treatment outcomes. The identification of patients at high risk of developing BM facilitates the optimization of treatment strategies and the consideration of PCI to prevent the development of BM. In this study, to enhance the prediction of the BM risk of NSCLC patients, we investigated and developed a deep learning-based segmentation and CT radiomics-based ensemble learning model. Our experimental results demonstrated that fusing CT radiomics and clinical features was feasible to improve the performance in terms of predicting the BM risk of NSCLC patients. The radiomics–clinical fusion model proved effective in predicting both BMFS and OS, as well as stratifying advanced NSCLC patients into high and low BM risk groups, which indicated the value of CT radiomics and clinical features in prognosis prediction. There were numerous characteristics of our study.

First, we fused CT radiomics and clinical features to develop a machine learning-based model to predict the BM risk of advanced NSCLC patients. In comparison with the CT radiomics-based and clinical feature-based models, the fused feature-based model improved the prediction performance in terms of predicting the BM risk of advanced NSCLC (P < 0.05 for the training cohort and validation cohort 1, Delong test). CT radiomics and clinical factors provided complementary information for predicting the BM risk of advanced NSCLC patients. By testing the clinical usefulness with the DCA method, the fused feature-based model also performed better than the individual feature-based model (as shown in Fig. 5). The CT radiomics model achieved significantly higher performance in terms of predicting the BM risk (P < 0.05 for all cohorts, Delong test) compared with that of the clinical feature-based prediction model. Thus, CT radiomics features provide more information than conventional clinical factors in BM risk prediction.

Second, to develop the BM risk prediction model, we proposed an ensemble learning method based on the XGBoost classifier to train and build the model. In this study, the ensemble learning approach based on the XGBoost classifier used a sequence of decision trees to improve the predictive performance. Compared with the performance of the other machine learning classifiers, i.e., the SVM, MLP and decision tree classifiers, the proposed ensemble learning model achieved the highest performance by using CT radiomics features (as shown in Fig. 4 (a)-(c) and Table 2). These results indicated that using ensemble learning methods, such as the XGBoost classifier, can potentially improve the predictive performance of a risk prediction model for BM. Thus, in future studies, this approach may be applied to other cancer types and clinical scenarios to improve the predictive performance.

Third, we initially computed 1106 noninvasive radiomics features to decode the CT imaging phenotypes of advanced NSCLC patients. Since the quality of radiomics features highly depends on the tumor segmentation accuracy, we developed a deep residual U-Net network to segment each lung tumor anatomically. To select robust radiomics features, we removed redundant features by using three feature selection steps involving the intraclass correlation coefficient (ICC) threshold, variance threshold, and LASSO-based RFE feature selection. To visualize the selected radiomics features, we used a voxel-based feature visualization technique. Radiomics refers to the extraction of quantitative features from primary lung tumors that can provide additional information beyond what is visible to the naked eye (as shown in Fig. 3 (c)). Since the development of BM is associated with changes in the primary lung microenvironment, these features can be used to characterize the tumor microenvironment and predict the BM risk of advanced NSCLC patients.

Fourth, we evaluated the prognostic value of the prediction scores generated by the fused feature-based model for predicting BMFS and OS. We hypothesized that the prediction scores may also be predictive of BMFS and OS, as the development of BM can be a significant factor in the prognosis of advanced NSCLC. To test this hypothesis, we conducted survival analysis using the prediction scores generated by the fused feature-based model as a predictor variable. The results showed that the prediction scores generated by the fused feature-based model were significantly associated with BMFS and OS (as shown in Fig. 6; Table 4). This suggests that the fused feature-based model may provide additional prognostic information beyond what can be obtained from conventional factors alone.

Despite the promising results of our study, there are some limitations that need to be considered. First, this was a retrospective study with a relatively small dataset. A small dataset can lead to overfitting, where the model learns noise in the data rather than true patterns. Therefore, our model needs to be validated using a larger, diverse dataset to ensure that the results are robust and applicable to a broader population. Meanwhile, a prospective study can provide a more rigorous validation of the model performance in a real-world clinical setting, as it can capture a wider range of patient characteristics and clinical scenarios. Therefore, a prospective study is needed to confirm the utility of the ensemble learning approach in clinical practice and to evaluate its potential for improving patient outcomes.

Second, only CT radiomics and clinical factors were used to develop the prediction model. The “seed-and-soil” theory of BM suggests that the successful formation of metastases in distant organs requires not only tumor cells with metastatic potential (the “seed”) but also a hospitable microenvironment in host organs (the “soil”) [24]. Previous studies have provided evidence that baseline brain magnetic resonance imaging can contribute to the prediction of BM risk in NSCLC patients [25, 26]. Thus, brain magnetic resonance imaging (MRI) also needs to be used to improve the performance of BM risk prediction. While other clinical data, such as positron emission tomography (PET), genetic or molecular data, may provide useful information, we also need to integrate these data to improve the model performance in future studies.

Third, there was a lack of uniformity in both the CT scanners and the associated acquisition parameters. This variation is particularly significant as CT radiomics feature computation relies heavily on consistent image quality. Despite implementing image resampling techniques to standardize CT images, the inherent diversity arising from different imaging parameters remains unavoidable. Moreover, only 1106 radiomics features were extracted in this study, which may not be sufficiently comprehensive to fully capture the underlying heterogeneity and complexity of the tumor microenvironment. Thus, efficient image standardization algorithms and more robust radiomics features should be explored in future studies.

Finally, we developed an ensemble learning model based on the XGBoost classifier, which may not be optimal. Although the XGBoost classifier yielded higher performance in comparison with other machine learning classifiers, it may not be the optimal classifier for ensemble learning. In addition to traditional machine learning algorithms and ensemble methods, deep learning methods, such as chat generative pretrained transformer (chatGPT) may also be explored to improve the performance of BM risk prediction models. Therefore, we should explore and develop more robust algorithms to facilitate the translation of the models into clinical practice.

**Conclusion**.

In this study, we developed a deep learning-based segmentation and CT radiomics-based ensemble learning model to enhance BM risk prediction within three years for advanced NSCLC patients. Applying a deep residual U-Net model, each lung tumor was segmented automatically and accurately. By fusing CT radiomics and clinical features, our proposed model improved the prediction performance in terms of predicting BM risk. Meanwhile, the radiomic-clinical fusion model also had prognostic value in predicting the BMFS and OS of NSCLC patients. Thus, based on the promising results, this study provided new evidence to support more research efforts focusing on developing optimal machine learning models to combine different types of phenotype features to predict the BM risk of NSCLC patients.

## Data Availability

The datasets used or analyzed during the current study are available from the corresponding author on reasonable request.

## List of Abbreviations

BM: Brain metastasis
NSCLC: Non-small cell lung cancer
KM: Kaplan-Meier
BMFS: Brain metastasis free survival
OS: Overall survival
PCI: Prophylactic cranial irradiation
CT: Computed tomography
LA-NSCLC: Locally advanced NSCLC
LoG: Laplacian of Gaussian
RFE: Recursive feature elimination
Lasso: Least absolute shrinkage and selection operator
SMOTE: Synthetic minority over-sampling technique
ROC: Receiver operating characteristic
AUC: Area under the ROC curve
CI: Confidence interval
ACC: Accuracy
SEN: Sensitivity
SPE: Specificity
PPV: Positive predictive value
NPV: Negative predictive value
OR: Odds ratio
MCC: Matthews correlation coefficient
DCA: Decision curve analysis
C-index: Harrell’s concordance index
HR: Hazard ratio
SVM: Support vector machine
MLP: Multi-layer perceptron
